# Indocyanine green‐guided robotic‐assisted mesorectal lymphadenectomy for high‐risk T1 rectal carcinoma: Preliminary results of a prospective cohort study (IDEAL stage 2a)

**DOI:** 10.1111/codi.70554

**Published:** 2026-07-15

**Authors:** Philippe Rouanet, Nicolas Flori, Nabila Bouazza, Amina Houmada, Lakhdar Khellaf, Christophe Taoum

**Affiliations:** ^1^ Department of Surgical Oncology, Montpellier Cancer Institute (ICM) University of Montpellier Montpellier France; ^2^ Department of Supportive and Palliative Care, Montpellier Cancer Institute (ICM) University of Montpellier Montpellier France; ^3^ Department of Clinical Research and Innovation, Montpellier Cancer Institute (ICM) University of Montpellier Montpellier France; ^4^ Department of Pathology, Montpellier Cancer Institute (ICM) University of Montpellier Montpellier France

**Keywords:** colorectal cancer, indocyanine green, lymphadenectomy, organ‐preserving surgery

## Abstract

**Aim:**

Lymph node metastases occur in 10%–15% of high‐risk T1 rectal tumours. Total mesorectal excision remains the standard treatment, whereas chemoradiotherapy is reserved for patients with frailty. Indocyanine green (ICG)‐guided lymphadenectomy may represent an organ‐preserving alternative for patients at high risk of node metastasis.

**Methods:**

From May 2021 to July 2025, 11 patients underwent curative transanal excision surgery for early‐stage low rectal cancer that was classified as high‐risk pT1 adenocarcinoma. ICG (1 mg) was injected submucosally to label all lymph nodes irrespective of their metastatic status. ICG‐guided robotic‐assisted mesorectal lymphadenectomy was performed with the Firefly Imaging System (INTUITIVE©).

**Results:**

Initially, ICG was injected intraoperatively (*n* = 2 patients). However, due to unsuccessful mapping in the second patient, ICG was then administered the day before surgery in all subsequent patients, resulting in successful visualisation and excision. Lymphatic drainage was not along the superior rectal artery in two patients. Lymphadenectomy was mainly performed along the superior rectal artery to its bifurcation into the right and left rectal arteries in the mid‐rectum. The median number of excised lymph nodes was 16.7 (range: 9–29). Lymph node invasion was detected in one patient who then received adjuvant chemotherapy. The median hospital stay was 2 days, without complications. After 36 months of follow‐up (range: 12–60), all patients were alive without disease progression or functional impairment.

**Conclusion:**

In patients with early T1 rectal tumours, ICG‐guided robotic‐assisted mesorectal lymphadenectomy seems to be technically feasible. These preliminary results, feasibility and oncological safety need to be confirmed in a prospective multicentre trial.

**What does this paper add to the literature?**

Indocyanine green‐guided robotic‐assisted mesorectal lymphadenectomy for high‐risk T1 rectal carcinoma appears technically feasible in selected patients. In this preliminary study, acceptable short‐term safety (median number of harvested lymph nodes = 16.7) was observed, while atypical lymphatic drainage outside the mesorectum was also identified.

## INTRODUCTION

The incidence of rectal cancer has increased in the past two decades. Every year, more than 700 000 new cases and approximately 300 000 deaths are reported worldwide [1]. The 5‐year relative survival has much improved, from 38% before 2000 to ~65% in recent years, largely due to screening programmes that facilitate the detection of early‐stage T1 tumours. Planned organ‐preserving strategies have demonstrated their efficacy in selected patients with small T2 and early T3 tumours [2]. However, the current guidelines continue to recommend total mesorectal excision (TME) for patients with high‐risk pT1 tumours (depth of submucosal invasion = sm2–3, tumour size > 3 cm, and tumour budding ≥ 2, with or without lymphovascular invasion) because they are associated with a risk of lymph node involvement of 10%–20% [3, 4]. Accurate local staging remains challenging; pelvic magnetic resonance imaging (MRI) and transanal ultrasound display sensitivities lower than 70% and limitations for predicting the lymph node status [5]. Therefore, standard TME, especially with coloanal anastomosis or abdominoperineal excision, may represent a substantial overtreatment in some patients, supporting the exploration of alternative strategies. For instance, the ongoing phase III TESAR trial compares local excision followed by adjuvant chemoradiotherapy and standard completion TME in patients with early‐stage rectal cancer [6]. Despite these efforts, new organ‐preserving strategies to adequately address the risk of lymph node metastasis are required. Perioperative lymphatic flow mapping and targeted lymphadenectomy may represent a promising alternative.

Indocyanine green (ICG), a near‐infrared fluorophore that is visualised using dedicated imaging systems, is used for tissue perfusion assessment, lymph node mapping [7], vital anatomic structure identification, and tumour tissue imaging [8]. Moreover, there are optimised protocols for the preoperative endoscopic submucosal injection of ICG to map lymph nodes [9].

In this study, we evaluated ICG‐guided robotic‐assisted mesorectal lymphadenectomy (RMRL) as an alternative strategy to assess lymph node involvement and to preserve the rectal anatomy and function.

## METHODS

### Study population

This prospective pilot study was carried out at Montpellier Cancer Institute (ICM), France, between May 2021 and July 2025. The protocol was registered in the French Health Data Hub (registration number: F20231109093052). It was initially approved by the ICM internal review board and then registered as an IDEAL protocol (ICM‐REG 2026/01) in 2026. This study followed the IDEAL stage 2a methodology and its objectives were to standardise the ICG‐guided RMRL protocol and to collect exploratory data on its effectiveness, feasibility, and safety (Supplementary Information) [10, 11].

### Inclusion criteria

Among all patients who underwent endoscopic resection (mucosal or submucosal dissection) or transanal surgical excision for early rectal cancer (N0 status confirmed by MRI before surgery), only individuals with high‐risk histological features (i.e. tumour budding, poor differentiation, lymphatic and/or vascular invasion), according to the French practice guidelines [4], were included. Specifically, the post‐operative pathological analysis confirmed the curative local resection (R0), and identified the excised tumours as high‐risk pT1 adenocarcinoma due to the following histological features: depth of submucosal invasion = sm2‐3, tumour size > 3 cm, and tumour budding ≥ 2 with or without lymphovascular invasion. In accordance with the French and US guidelines [3, 4], transabdominal completion TME was recommended at the multidisciplinary team (MDT) meeting. RMRL was proposed as an alternative strategy. Indeed, all cases were discussed at MDT meetings, and treatments were decided on an individual basis following detailed discussion with each patient. Patients were explicitly informed that if lymphatic mapping by ICG was unsuccessful, the standard management (i.e., TME) would be carried out. The rationale, potential benefits, risks and alternative treatment options were thoroughly discussed with each patient before the procedure. Patient information and treatment decision‐making were documented in the medical records and MDT reports, and shared with the referring physicians, ensuring full traceability of both the decision‐making process and informed consent.

### Indocyanine green injection protocol

All patients underwent mechanical bowel preparation on the day before RMRL. Endoscopic submucosal tattooing was performed. Circumferential injection of ICG (Infracyanine® 25 mg/1 mL–SERB© Pharmaceuticals) was performed at two sites around the surgical incision (0.2 mL per injection, total dose 1 mg), according to a protocol adapted from Ahn et al. [9]. Following injection, ICG diffuses through the lymphatic system and accumulates in macrophages within the lymph nodes, allowing the visualisation of the lymphatic flow, regardless of metastatic involvement. Therefore, it gives information on the lymphatic system anatomy rather than on tumour dissemination. In the first two patients, ICG was administered intraoperatively. As adequate lymph node staining was not achieved in the second patient, the protocol was modified, and ICG was injected 14 h before RMRL in all the other patients.

### Operative technique

RMRL was performed by the senior author (PR, 800 robotic TMEs already performed), using the Da Vinci XI™ robotic surgical system (Intuitive Inc.). The procedure began with the laparoscopic exploration of the peritoneal cavity. The Firefly Imaging System allows real‐time visualisation of lymphatic vessels and lymph nodes.

Lymph node sampling began at the root of the inferior mesenteric artery, followed by dissection along the fluorescent lymphatic vessels and nodes. Lymphatic vessels were not routinely excised. The mesorectum was opened at the level of the sacral promontory. In most patients, lymph nodes were excised along the superior rectal artery to its bifurcation into the right and left rectal arteries in the mid‐rectum. All fluorescent green lymph nodes were removed, with careful verification that no residual fluorescence remained. Lastly, lymph nodes at the rectal wall areas where ICG was injected (characterised by intense green fluorescence) were also removed. Particular care was taken to avoid rectal wall injury. Drainage was not routinely required because the procedure did not cause bleeding. The total number of harvested lymph nodes and of metastatic lymph nodes was recorded. Specimens submitted for pathological examination were systematically labelled according to their anatomical location at harvesting time. Specimen localisation was based on predefined anatomical landmarks identified intraoperatively (i.e., inferior mesenteric artery, sacral promontory, superior rectal artery) to ensure consistent topographical assignment of all harvested lymph nodes. Following lymph node dissection, the histological results were discussed at a new MDT meeting during which the management of patients with lymph node involvement was determined based on the extent of nodal disease and the patient characteristics. The protocol recommended adjuvant chemotherapy for patients with limited node involvement (< 3 positive lymph nodes) and salvage TME for patients with more extensive nodal disease.

### Follow‐up

All patients underwent standardised follow‐up: rectal examination and MRI every four months during the first year and every six months for four years.

### Statistical analysis

Data were collected and analysed using Microsoft Excel (Microsoft Corp., Redmond, WA, USA). Descriptive statistics were used to summarise the data; inferential analyses were not performed given the limited sample size.

## RESULTS

Eleven of the 150 patients treated annually for rectal cancer at Montpellier Cancer Institute underwent ICG‐guided RMRL. This low number of patients with early‐stage disease reflects our role as a referral centre for patients with locally advanced rectal cancer. Four of the 11 patients were men, the median age was 61.2 years (34–73), the mean body mass index was 29.5 kg/m^2^ (50% of patients had obesity), and the American Society of Anaesthesiologists (ASA) Physical Status Classification System score was ≥ 2 in 10/11 patients.

All tumours were identified as adenocarcinoma without microsatellite instability (pMMR). The median tumour size was 31 mm (range: 20–50), 60% were classified as sm3 with tumour budding = 2–3; (budding = 1 associated with vascular invasion in one tumour). All tumours were located in the lower third of the rectum. The mean distance between the lower tumour margin and the levator ani muscle was 12 mm (range: 0–40). The final pathological staging was pT1 for all 11 patients. Primary tumour resection (endoscopic or transanal surgical) was considered curative, and margins were negative (R0). The patient and tumour characteristics are summarised in Table 1.

**TABLE 1:** Patient and tumour characteristics.

Patient	Sex	Age (years)	BMI	ASA	Comorbidities	Resection	Tumour size (cm)	pT	Sm	Budding	Emboli	Dissected lymph nodes (*n*)	pN+ lymph nodes (*n*)
1	Male	66	31	2	HBP and D	M	3	1	3	2	no	9	0
2	Male	71	32	2	no	M	2	1	2	3	no	22	0
3	Female	34	25	1	no	TEM	4	1	2	1	Vx	29	1
4	Female	62	25	2	Breast cancer	M	2	1	2	2	no	11	0
5	Female	50	24	2	no	TEM	3	1	2	2	no	18	0
6	Male	69	30	2	HBP and D	TEM	3	1	3	2	no	15	0
7	Female	73	35	2	Lymphoma B	TEM	3	1	2	2	no	12	0
8	Female	50	31	2	no	TEM	3	1	3	3	no	14	0
9	Male	72	31	3	AF	TEM	3	1	3	2	no	18	0
10	Female	65	26	2	no	TEM	3	1	3	2	no	17	0
11	Female	58	35	2	no	TEM	5	1	2	2	no	16	0

Abbreviations: AF, atrial fibrillation; ASA (American Society of Anesthesiologists) Physical Status Classification System; BMI, Body Index Mass; D, diabetes; HBP, high blood pressure; M, mucosectomy; N+, positive lymph node; TEM, transanal endoscopic microsurgery; Vx, vascular.

The mean number of harvested lymph nodes was 16.8 (range: 9–29). One positive node was identified in a 34‐year‐old woman (1/29 lymph nodes) who had a 4 cm tumour with depth of submucosal invasion = sm2, budding grade 1 and vascular invasion. The positive lymph node was isolated and located in the peritumoral area, without any evidence of additional nodal disease. The MDT discussed risks and benefits of the available treatment options and recommended 3 months of adjuvant FOLFOX chemotherapy, in accordance with the protocol guidelines. This recommendation also aligned with the patient's strong desire for future pregnancy and fertility preservation. The patient remains disease‐free after 4 years of follow‐up and achieved a successful pregnancy.

**FIGURE 1:**
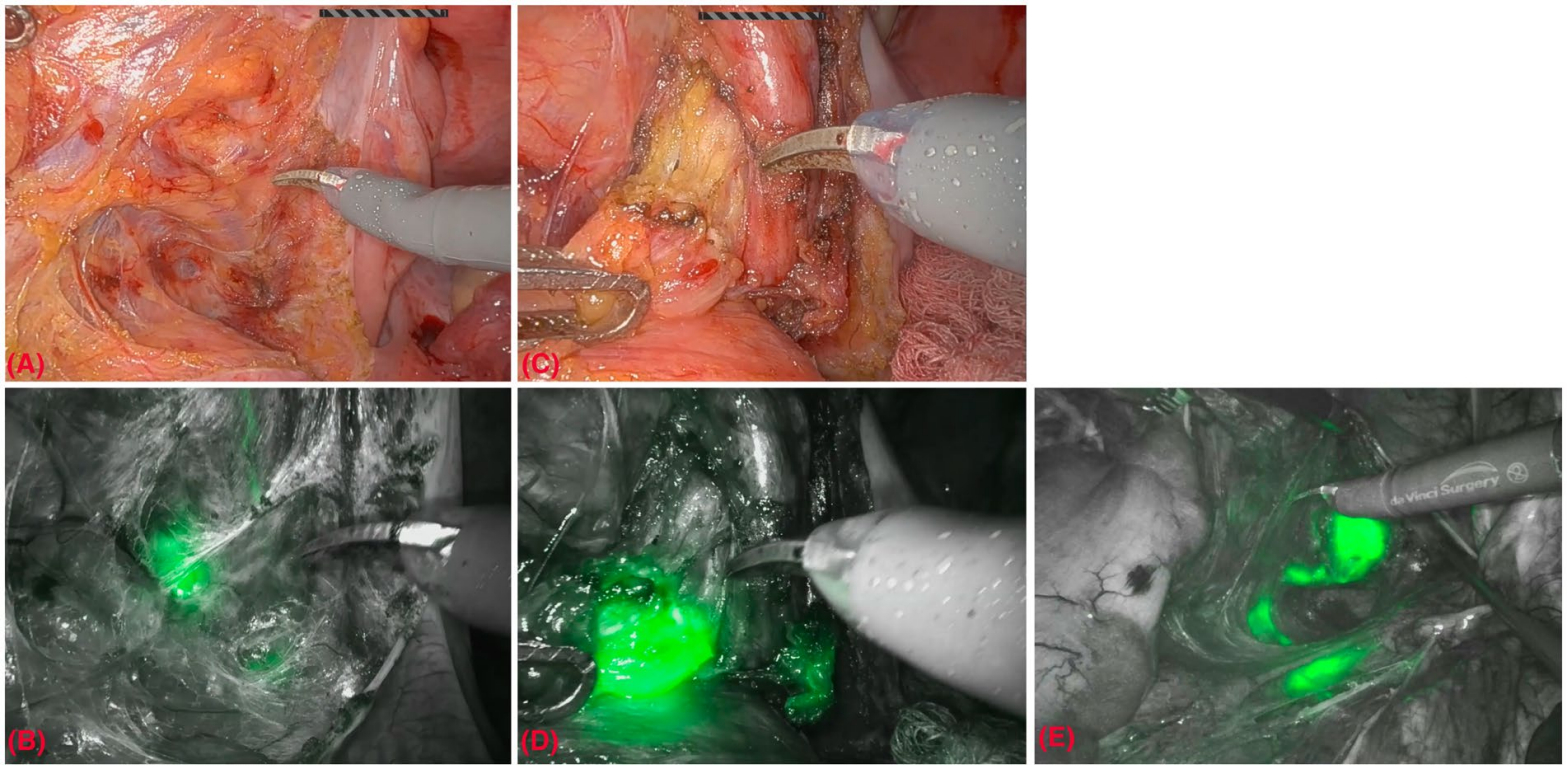
Representative examples of lymph node harvesting. (A, B) Example of lymph node poorly visualised under white‐light imaging (A), but readily identified using indocyanine green fluorescence imaging (B). (C, D) Representative example of lymph node retrieval adjacent to the superior rectal artery under white‐light imaging (C) and with indocyanine green fluorescence imaging (D). (E) Example of atypical retro‐mesorectal lymphatic drainage, demonstrating cross‐over from a lymph node along the left middle rectal artery across the sacrum, visualised using indocyanine green fluorescence imaging.

Adequate lymph node visualisation was achieved in 10/11 patients (91%), and in nine of them, ICG was administered the day before surgery (Figure 1). During the intervention, the surgeon could alternate between standard visible light and near‐infrared imaging. Selective lymph node sampling was performed with meticulous preservation of the vascular and nervous structures, and all ICG‐positive lymph nodes were harvested. Non‐mutilating and precise dissection was facilitated by the robotic camera stability and the wristed instrument articulation. The fluorescent lymph nodes could not be visualised in one male patient (patient #2; body max index = 32 kg/m^2^) in whom ICG was injected intraoperatively. Therefore, the procedure was converted to complete TME with coloanal anastomosis. No residual tumour was identified in the specimen, and all 22 harvested lymph nodes were negative. Two patients with ultra‐low rectal tumours had atypical lymphatic drainage towards the lateral pelvic compartment, outside the conventional mesorectal artery drainage area. Drainage occurred via the medial rectal artery. ICG diffusion extended into the pelvic side wall, retro‐mesorectal space, and above the sacral promontory towards the lateral aortic region.

For the 10 patients with successful ICG‐guided lymphadenectomy, the median hospital stay was 2 days, without post‐operative complications. After a median follow‐up of 36 months (range 12–60), all patients were alive, without evidence of disease progression or functional impairment.

## DISCUSSION

This preliminary prospective IDEAL stage 2a study suggests that ICG‐guided RMRL is a feasible organ‐preserving approach that may represent an alternative to standard TME or experimental chemoradiotherapy in selected patients with high‐risk pT1 rectal tumours. The high number of harvested lymph nodes and the absence of post‐operative and medium‐term morbidity in this challenging cohort with ultra‐low tumours support further prospective investigation.

### ICG injection protocol

ICG is a useful pre‐operative tattooing agent that emits fluorescence under near‐infrared light, with a maximum peak wavelength of approximately 830 nm. When injected into the subserosal or submucosal layers, ICG diffuses through the lymphatic system and binds to macrophages within lymph nodes, allowing lymph node mapping. Considerable variability (injection method and ICG dosage) exists among tattooing protocols. Ahn et al. [9] evaluated different ICG concentrations, number and timing of injections. They concluded that optimal lymph node visualisation requires the pre‐operative endoscopic submucosal injection at least at two sites of a total ICG dose between 0.5 and 1 mg. In our study, a standard dose of 1 mg was used in all patients. RMRL could be carried out in all patients who received the ICG injection on the day before surgery, but not in one of the two patients in whom ICG was administered during surgery, due to poor lymph node visualisation. This suggests that pre‐operative injection may enhance lymph node visualisation, at least for rectal cancer.

### Sentinel node technique in early rectal cancer

The sentinel node technique could be more relevant in patients with early‐stage rectal cancer than in those with advanced disease where lymphatic obstruction, caused by tumour infiltration, may alter the physiological lymphatic flow. However, data on sentinel nodes in rectal cancer are limited. Handgraaf et al. were among the first to analyse the feasibility of ICG mapping of sentinel nodes with inconclusive results [12]. In theory, in the mesorectal space, lymphatic flows should be in a bottom‐up direction. Ammirati et al. found that most harvested lymph nodes are located within the same quadrant as the primary tumour and that the distance between the first metastatic lymph node and the tumour rarely exceeds 3 cm [13]. Importantly, sentinel lymph node identification in early rectal carcinoma lacks standardisation, and currently, it is not applicable in routine clinical practice, reinforcing the oncological rationale for complete lymphadenectomy.

### Atypical drainage and lateral lymph node dissection

The prospective GREENLIGHT trial assessed real‐time lymph node visualisation in colorectal cancer following pre‐operative ICG injection [14]. Among the patients with rectal tumour, the planned extent of lymphadenectomy was modified in 46.7% of patients (*n* = 7/12) who underwent upfront surgery and in 26.3% of patients (*n* = 5/19) after neoadjuvant treatment (*p* = 0.130). This was mainly explained by the identification of lymph nodes outside the standard draining area, particularly in the right para‐aortic area. Moreover, ICG fluorescence can facilitate lateral pelvic lymph node dissection, resulting in a greater yield [15].

### Breaching the mesorectum

Since the 1980s, TME has been considered the gold standard for curative rectal cancer surgery [16]. Breaching the mesorectum envelope is considered inadequate and associated with higher local recurrence rates [17]. In the 1980s, peri‐rectal lymphadenectomy was described in early attempts at organ preservation [18]. Most studies on extra‐lymphatic diffusion in the mesorectal area focused on advanced cancers. In pT1 disease, only nodal involvement was reported. Therefore, the safety of opening the mesorectum envelope for selective lymphadenectomy in pT1 tumours may be questioned, especially due to the risk of extra‐lymphatic tumour dissemination. A retrospective analysis conducted by the French Research Group on Rectum Surgery (GRECCAR) using data from 425 pathology reports of pT1 tumours after TME revealed a node positivity rate of 14.6%, predominantly in patients with sm3 lesions (unpublished data). Overall, tumour deposits were identified in 1.8% of cases and venous emboli in 5.3%. In node‐negative patients, tumour deposits were rare (0.3%) and venous emboli were observed in 5.3%. In node‐positive patients, tumour deposits were observed only in patients with ≥ 3 positive lymph nodes. Although these data require confirmation, they suggest that the risk of extra‐lymphatic dissemination is very low in patients with pT1N0 tumours, especially in the absence of vascular emboli.

### Safety of ICG‐guided RMRL

In this study, no patient required completion TME following lymph node dissection. Postoperative MRI confirmed that the mesorectal anatomy was preserved, without evidence of significant fibrosis, distortion of the mesorectal planes, or other sequelae likely to compromise a subsequent TME.

Although any pelvic dissection may induce scarring and increase the technical complexity of subsequent surgery, the lymph node dissection performed in this study was limited in extent and minimally invasive. Consequently, we consider it plausible that completion TME, if required, would remain feasible. As no patient in this series underwent completion TME following RMRL, this assumption cannot be confirmed and warrants evaluation in future studies.

Few studies reported combined local excision and lymphadenectomy, most commonly using transanal endoscopic microsurgery and local lymphadenectomy [19, 20]. Recently, Benzoni et al. demonstrated the feasibility of fluorescence‐guided mesorectal lymph node harvesting combined with local excision of early‐stage rectal cancer [21]. Nonetheless, they reported a failure rate of 20% in lymph node visualisation and a median harvest of 6.8 nodes (range: 0–15). Conversely, with our standardised protocol (1 mg ICG administered 14 h before surgery), the mean number of harvested lymph nodes was 16.8 (range: 9–29). The procedure seems to be safe and reproducible, without post‐operative complications and good organ preservation. Although all patients met the criteria for completion TME, lymph node metastasis was detected only in one patient (9%), in agreement with the expected rate for high‐risk pT1 rectal tumours. Organ preservation was achieved in all patients, whereas colorectal anastomosis would have been required in ~70% of them with standard completion TME. These outcomes compare favourably with the abdominoperineal resection rate of 45% after completion TME by transanal endoscopic microsurgery [22].

## CONCLUSION

This preliminary study suggests that RMRL is a feasible procedure for the targeted removal of anatomically mapped, ICG‐positive mesorectal lymph nodes, with minimal postoperative morbidity and encouraging short‐term oncological outcomes. However, the limited sample size restricts the strength of the evidence and precludes definitive conclusions. Larger prospective multicentre studies are required to confirm the oncological safety of selective mesorectal opening in pT1 disease, to better characterise the clinical significance of atypical lymphatic drainage patterns, and to validate the lymph node yield and long‐term outcomes. This organ‐preserving strategy seems promising for young patients wishing to avoid radiotherapy, and for patients with ultra‐low rectal tumours in whom conventional radical surgery is associated with substantial functional morbidity.

## AUTHOR CONTRIBUTIONS

**Christophe Taoum:** Investigation; resources; writing – review and editing. **Philippe Rouanet:** Investigation; resources; data curation; formal analysis; visualization; writing – original draft; writing – review and editing. **Amina Houmada:** Investigation; resources; writing – review and editing. **Lakhdar Khellaf:** Investigation; resources; writing – review and editing. **Nicolas Flori:** Investigation; writing – review and editing; resources. **Nabila Bouazza:** Project administration; writing – review and editing.

## FUNDING INFORMATION

The authors have nothing to report.

## CONFLICT OF INTEREST STATEMENT

PR is a Proctor for Intuitive. All other authors declare no competing interests.

## ETHICS STATEMENT

This study was registered in the French Health Data Hub (registration number: F20231109093052) and was approved by the Montpellier Cancer Institute internal review board (ICM‐REG 2026/01). All patients approved the design and agreed to share their decision‐making procedure.

## Supporting information


**Data S1.** Reporting Guideline Checklist for IDEAL Stage 2a: Development.

## Data Availability

The anonymized data may be made available from the corresponding author upon reasonable request and with permission.
