# Supplementary material for: Indocyanine green‐guided robotic‐assisted mesorectal lymphadenectomy for high‐risk T1 rectal carcinoma: Preliminary results of a prospective cohort study (IDEAL stage 2a)
Source: Colorectal Dis. 2026 Jul 15;28(7):e70554. doi: 10.1111/codi.70554 (PMC13373332; doi:10.1111/codi.70554)
Supplement: Supplementary file 1 — Data S1. Reporting Guideline Checklist for IDEAL Stage 2a: Development. [file CODI-28-0-s001.pdf]

Reporting Guideline Checklist for IDEAL Stage 2a: Development

Section/Topic	Item No	Checklist Item	Reported on Page No
**Title and Abstract**			
Title and Abstract	1a	Identification as a prospective case series of a novel technique in the title, including the IDEAL stage in the title or abstract	1
	1b	Provide a structured summary of background, methods, results, and conclusions	3
**Introduction**			
Background and objectives	2a	Review of existing scientific literature, including reference to IDEAL Stage 1 reports in previous publications, if applicable	5
	2b	Specific objectives stated, including refining the technique and progressing toward stability	5-6
**Methods**			
Design	3	Description of study design (e.g. sequentially reported prospective case series)	6

Participants	4a	Detailed account of patient inclusion and exclusion criteria	6 - 7
	4b	Informed consent process described, including explanation of risks and acknowledgement of level of experience with technique/device	6 - 7
	4c	Setting, location, and timeframe of when and where the novel technique was performed, including hospital characteristics and appropriate details regarding the operator/team (e.g. experience)	6 - 7
Intervention	5a	Clear and detailed description of (or reference to) planned technique, including necessary pre-operative and post-operative care	7 - 8
	5b	Patient safety monitoring methods and safeguards	8
Outcomes	6	Description of outcome measure(s) selected and how they will be assessed, including patient reported outcome measures, when appropriate, utilising those measures that are standardised and validated, when available and applicable. When these are not available, provide rationale for the outcome measure(s) used	8
**Results**			
Baseline data	7	Baseline demographic and clinical characteristics for each patient. Include how many patients were assessed for treatment and a description of which patients were included, excluded, or refused, and why (to be displayed in a flow diagram format, when appropriate)	8 - 10<br>+ Table 1
Intervention	8	Transparent reporting of all cases in the sequence they were performed, clearly indicating when and why modifications to the technique took place, including visual aids of the technique and modifications (e.g. photographs, videos, etc) when available	9 - 10

Outcomes	9	Clinical and patient-reported outcomes described for each patient, with all available outcome data incorporated into a comprehensive table or graph, whenever possible, to allow for the relationship to be clearly visualized between technique modifications and outcomes	8 - 10<br>+ Table 1
Harms	10	Transparent account of all harms or unintended effects reported for each patient	9 - 10
**Discussion**			
Interpretation	11	Analysis of technique development, including consistency of results and a balanced discussion of benefits and harms	10 - 13
Limitations	12	Study limitations, addressing sources of potential bias	10 - 13
Stage End-Points	13a	Have the technique and outcomes reached stability in the hands of the current team (e.g. there is no intent to make further major modifications to the technique, and patient outcomes are stable)? Include an explanation of how you determined stability	10 - 13
	13b	Discussion of whether the technique is ready for evaluation in a prospective, multi-centre IDEAL Stage 2b study, and identification of indications for the technique	10 - 13
Conclusions	14	Conclusions and relevance, including plans to progress to future IDEAL stages, if applicable	13
**Other information**			
Protocol	15	Please quote reference or DOI if a protocol was written in advance and made available. If a protocol was not made available, consider including as a supplement if the journal allows	N/A

Ethics	16	Reference to ethical approvals obtained, and independent oversight, if applicable	2 and 6
Funding	17	Sources of funding and support, role of funders, and other conflicts of interest	N/A
Regulatory Approvals	18	Regulatory approvals being sought or obtained (e.g. CE Marking, FDA approval, etc) including the date of approval, if applicable	2 and 6

Bilbro NA, Hirst A, Paez A, et al. The IDEAL Reporting Guidelines: A Delphi Consensus Statement Stage specific recommendations for reporting the evaluation of surgical innovation [published online ahead of print, 2020 Jul 7]. Ann Surg. 2020;10.1097/SLA.0000000000004180. doi:10.1097/SLA.0000000000004180
